# Life in the London Slums

**Published:** 1882-02

**Authors:** 


### LIFE IN THE LONDON SLUMS.

A ghastly picture of life in the London slums was unfolded before the Southwark coroner yesterday at an inquest on a six-year-old girl who was killed by tripping in a hole in the rotten staircase of a house in 42 Gunn street. The child's mother, who is the wife of a hard-working general dealer and hawker, said she had asked the landlord to mend the stairs, but the work had not been done. The house, said the doctor, was in a shocking condition, not fit for any human being to live in. He knew the family to be straightforward, decent people, who paid five shillings a week for two abominable rooms which were not worth one-fifth that rent. The mother deposed that she herself had tumbled through the floor, and a short time since three of her children lay dead at one time with the measles, and the ceiling was so rotten that it fell down in lumps upon the coffins, and had never been mended yet. The measles, said the doctor, were the result of the bad sanitary state of the house. The jury returned a verdict of accidental death, with a rider censuring the landlord. Some honest folk will think that it would have been more to the point if they had returned a verdict of manslaughter against the landlord, his agent and the sanitary inspector of the district.—*Pall Mall Gazette.*

A BRIGHT youth, undergoing examination a few days since for admission to one of the departments at Washington, found himself confronted with the question:

"What is the distance from the earth to the sun?"

Not having the exact number of miles with him, he wrote in reply:

"I am unable to state accurately, but don't believe the sun is near enough to interfere with a proper performance of my duties if I get this clerkship."

He got it.

